# Ginseng intake and Alzheimer disease-specific cognition in older adults according to apolipoprotein **ε**4 allele status

**DOI:** 10.3389/fnagi.2023.1152626

**Published:** 2023-04-12

**Authors:** Boung Chul Lee, Young Min Choe, Guk-Hee Suh, Ihn-Geun Choi, Hyun Soo Kim, Jaeuk Hwang, Dahyun Yi, Jin Hyeong Jhoo, Jee Wook Kim

**Affiliations:** ^1^Department of Psychiatry, Hallym University College of Medicine, Chuncheon, Gangwon, Republic of Korea; ^2^Department of Neuropsychiatry, Hallym University Hangang Sacred Heart Hospital, Seoul, Republic of Korea; ^3^Department of Neuropsychiatry, Hallym University Dongtan Sacred Heart Hospital, Hwaseong, Gyeonggi, Republic of Korea; ^4^Department of Psychiatry, Seoul W Psychiatric Office, Seoul, Republic of Korea; ^5^Department of Laboratory Medicine, Hallym University Dongtan Sacred Heart Hospital, Hwaseong, Gyeonggi, Republic of Korea; ^6^Department of Psychiatry, Soonchunhyang University Hospital, Seoul, Republic of Korea; ^7^Institute of Human Behavioral Medicine, Medical Research Center, Seoul National University, Seoul, Republic of Korea; ^8^Department of Psychiatry, Kangwon National University School of Medicine, Chuncheon, Gangwon, Republic of Korea

**Keywords:** ginseng, cognition, Alzheimer’s disease, apolipoprotein **ε**4, memory

## Abstract

**Background:**

The probable association among ginseng intake, Alzheimer’s disease (AD)-specific cognition, and apolipoprotein ε4 (APOE4) remains poorly investigated. Hence, we examined the association between ginseng intake and AD-specific cognition in older adults under the moderating effect of APOE4 status.

**Methods:**

This study enrolled 160 adults aged 65–90 years without dementia. All participants underwent comprehensive dietary and clinical assessments including ginseng intake, AD-related cognition (i.e., delayed episodic memory, as the earliest cognitive change in AD), and non-memory cognition for comparative purposes.

**Results:**

Ginseng intake was associated with higher delayed episodic memory, but not non-memory cognition, compared to no ginseng intake. The interaction between ginseng intake and APOE4 status had a significant effect on delayed episodic memory. Subgroup analyses showed that ginseng intake was associated with higher delayed episodic memory in the APOE4-negative but not the APOE4-positive subgroup. The benefits of ginseng intake on delayed episodic memory were prominent in the high duration (≥5 years) and midlife onset (<65 years) groups.

**Conclusion:**

Our study of older adults with no dementia suggests that ginseng intake (with high duration and midlife onset) had a beneficial effect on AD-specific cognitive decline, i.e., the delayed episodic memory. In addition, APOE4 status moderates the association between ginseng intake status and AD-specific cognitive decline.

## Introduction

Alzheimer’s disease (AD) is a chronic neurodegenerative disease that primarily affects memory-related cognitive performance in the elderly (Querfurth and LaFerla, 2010). The earliest cognitive change in AD is episodic memory decline, particularly in delayed episodic memory decline (Howieson et al., 1997; Grober et al., 2000; Laakso et al., 2000; Bäckman et al., 2001, 2005; Tromp et al., 2015). AD is one of the most detrimental diseases worldwide because no therapeutic drug is currently available. Apolipoprotein ε4 (APOE4), the highest-risk gene for AD (Yamazaki et al., 2019), affects the pathway serves to increase Aβ pathology in the brains of APOE4-positive older adults (Morris et al., 2010; Yamazaki et al., 2019), and it may reduce function in multiple brain homeostatic pathways thereby causing cognitive impairment and dementia (Lowe et al., 2014; Yamazaki et al., 2019; Jeon et al., 2020).

In this background, ginseng, a widely used traditional medicine for centuries, could be a new alternative in the treatment of AD. Ginseng has been reported to have protective effects *via* multiple brain pathways, including anti-oxidative, anti-inflammatory, and immunomodulatory properties (Park et al., 2013; Hossen et al., 2017), and some preclinical studies have revealed that ginseng has an inhibitory effect on Aβ pathology (Lee et al., 2001; Kim et al., 2013; Choi et al., 2017, 2020). Many human studies have also reported non-specific cognitive benefits of ginseng (Geng et al., 2010; Lho et al., 2018). However, few human studies have investigated the effect of ginseng on the AD-specific cognitive domain, i.e., delayed episodic memory (Howieson et al., 1997; Grober et al., 2000; Laakso et al., 2000; Bäckman et al., 2001, 2005; Tromp et al., 2015). In addition, it is probable that ginseng affects AD and cognitive dysfunction based on an individual’s APOE4 status, as APOE4 is the most important gene in AD (Yamazaki et al., 2019). However, some aspects of AD and the associated cognitive decline, including the interaction between ginseng and APOE4, remain unclear.

Therefore, we first investigated the association between ginseng and AD-specific cognition, i.e., delayed episodic memory decline, in non-demented older adults. We then examined the moderating effect of APOE4 status on this association and the association between ginseng and non-memory cognition for comparative purposes.

## Methods

### Participants

The present study enrolled 160 non-demented older adults (age 65 to 90 years); 93 cognitively normal (CN) adults and 67 with mild cognitive impairment (MCI). Subjects recruited in the study are adults who attended a dementia screening program offered by the memory clinic of Hallym University Dongtan Sacred Heart Hospital, Hwaseong, Republic of Korea. Volunteers were invited for an eligibility assessment. Additional volunteers were enrolled from the community through recommendations from other subjects, acquaintances, friends, or family members.

The CN group comprised subjects with a Clinical Dementia Rating (Morris, 1993) score of 0 and no diagnosis of MCI or dementia. All MCI subjects met the current consensus for amnestic MCI criteria, including memory complaints confirmed by an informant, objective memory problem, preservation of global cognitive performance, independence in functional activities, and the absence of dementia. In the objective memory impairment assessment, the age-, education-, and sex-adjusted z-score was < −1.0 on at least one of 4 episodic memory tests in the Korean version of the Consortium to Establish a Registry for Alzheimer’s Disease (CERAD) neuropsychological battery (Morris et al., 1989; Lee et al., 2002): Word List Memory (WLM), Word List Recall (WLR), Word List Recognition (WLRc), and the Constructional Recall (CR) test (Morris et al., 1989; Lee et al., 2002, 2004). All MCI subjects had a Clinical Dementia Rating score of 0.5. The exclusion criteria were the presence of a major psychiatric disorders, a significant neurological or medical illness, or a comorbidity that could affect cognitive functioning; illiteracy; visual/hearing difficulties or severe communication or behavioral problems that could make clinical examinations difficult; and the use of an investigational drug. The study protocol was approved by the Institutional Review Board of the Hallym University Dongtan Sacred Heart Hospital. The study was conducted it in accordance with the recommendations of the current version of the Declaration of Helsinki and all subjects have provided informed consent.

### Clinical assessments

All subjects underwent standardized clinical assessments by psychiatrists with more than 10 years of clinical experience based on the clinical assessment protocol, which incorporates the CERAD clinical and neuropsychological battery (Morris et al., 1989; Lee et al., 2002). Trained psychometrists administered the neuropsychological assessment protocol incorporating the CERAD neuropsychological battery (Lee et al., 2004) to all subjects. Memory (immediate or delayed episodic memory) and non-memory cognition scores were also measured. The episodic memory score (EMS) was determined by summing the scores of 4 episodic memory tests (WLM, WLR, WLRc, and CR). Immediate EMS was determined by the score of WLM. Delayed EMS, as the earliest cognitive change in AD (Howieson et al., 1997; Grober et al., 2000; Laakso et al., 2000; Bäckman et al., 2001, 2005; Tromp et al., 2015), was determined by summing the scores of 3 delayed episodic memory tests (WLR, WLRc, and CR). The non-memory score (NMS) was calculated by summing the scores of 3 non-memory tests (verbal fluency, modified Boston naming test, and constructional praxis). Vascular risks (e.g., hypertension, diabetes mellitus, dyslipidemia, coronary heart disease, transient ischemic attack, and stroke) were measured based on data collected by trained psychometrists during systematic interviews of the subjects and their families. The vascular risk score (VRS) was computed based on the number of vascular risk factors present and was reported as a percentage (DeCarli et al., 2004). The Geriatric Depression Scale (GDS) was used to assess the severity of depressive symptoms (Yesavage et al., 1982; Kim et al., 2008). Lifetime alcohol intake status (never/former/drinker) were evaluated by trained psychometrists interviews and a medical record review. The accuracy of the information was ensured by interviewing reliable informants.

### Assessment of ginseng intake and other dietary patterns

All subjects were assessed systematically to identify their ginseng intake patterns, i.e., ginseng type (white-or red-ginseng in any formula containing ginseng extract), the age at initial ginseng intake, and the frequency of ginseng intake (number of days per month).(Lho et al., 2018) As the quantity of ginseng varies between ginseng items and there is a low probability of obtaining the exact quantity of ginseng in any given item, we defined ‘ginseng intake’ by subtracting the age at initial ginseng intake from the age at study entry, adding 1 year to reflect the intake of the first year, and then multiplying the result by a year’s intake, calculated by multiplying the number of days of ginseng intake in a month by 12 months (Lho et al., 2018). As the median of the years of cumulative ginseng intake in subjects with ginseng consumption was 5 years, ginseng intake was categorized as ‘no use,’ ‘low use’ (< 5 years), or ‘high use’ (≥ 5 years).(Lho et al., 2018) We also assessed dietary patterns including food types, such as protein, fruit or vegetables, using the items from the mini nutritional assessment (MNA) tools, which is a brief, valid nutritional evaluation tool for elderly populations (Vellas et al., 1999).

### Blood test

Blood samples were obtained by venipuncture in the morning after an overnight fast. Albumin, glucose, and high-(HDL) and low-density lipoprotein (LDL)-cholesterol were measured using a Roche COBAS c702 analyzer and dedicated reagents (Roche Diagnostics, Mannheim, Germany).

### APOE genotyping

Blood samples were collected into vacutainer tubes with EDTA anticoagulant. DNA was extracted using QIAamp DSP DNA Blood mini kits (QIAGEN, Hilden, Germany) and the QIAcube HT System (QIAGEN). APOE genotyping was performed with the aid of a Seeplex ApoE ACE Genotyping Kit (Seegene, Seoul, Korea) and the ProFlex PCR system (ThermoFisher Scientific, Waltham, MA, USA) according to the manufacturers’ instructions. PCR products were analyzed *via* capillary electrophoresis (QIAxcel Advanced System, QIAGEN) and labeled ε2/ε2, ε2/ε3, ε2/ε4, ε3/ε3, ε3/ε4, or ε4/ε4 by the electrophoresis patterns as instructed by the manufacturer. APOE4-positivity was defined as the presence of at least one ε4 allele.

### Statistical analysis

We first compared the demographic and clinical characteristics by ginseng intake status (no intake vs. ginseng intake) using the Student t-test to analyze continuous data and the chi-square test or Fisher exact test to evaluate categorical data. To examine the relationship between ginseng intake and cognition, multiple linear regression analyses were performed with ginseng intake as the independent variable and cognition as the dependent variable. For these analyses, we used the “no ginseng intake” as the reference. As various factors may influence the association between ginseng intake and cognition, we systematically evaluated all subjects to identify potential confounders, such as vascular risks, depression, annual income, alcohol intake, smoking, blood nutritional markers (e.g., albumin, glucose, or HDL-and LDL-cholesterol), and dietary patterns including food types (e.g., protein, fruits, or vegetables). We tested two models, adjusting for the covariates in a stepwise manner. The first model included age, sex, APOE4 status, education, clinical diagnosis, VRS, GDS, and blood nutritional markers (e.g., albumin, glucose, or HDL-and LDL-cholesterol) as covariates; the second model included those covariates plus annual income status, alcohol intake, smoking and dietary patterns including food types (e.g., protein, fruits, or vegetables).

The moderating effect of APOE4 status on the association between ginseng intake and cognition was examined in multiple linear regression analyses including a two-way interaction terms for the association between ginseng intake and cognition as additional independent variables. When an interaction was determined, linear regression analyses were repeated individually depending on whether the APOE4 was positive or negative. To examine differential effects, the similar sensitivity analyses were performed using only the subjects with cognitive impairment because the cognitive score may be less discriminatory in CN individuals. In addition, the similar sensitivity analyses were performed using the subjects without decrease in food intake over the past 3 months. In order to eliminate possible effects of physical or mental conditions in relation to both ginseng intake or cognition, subjects with changes of food intake due to loss of appetite, digestive problems, and chewing or swallowing difficulties were excluded from the analyses. All statistical analyses were performed using SPSS Statistics ver. 28 (IBM Corp, Armonk, NY, United States).

## Results

### Participant characteristics

Table 1 summarizes the demographic and clinical characteristics of the 160 participants by ginseng intake status (80 who took ginseng and the 80 who did not). None of the study subjects were malnourished, i.e., serum albumin <3.5 g/dL (Cabrerizo et al., 2015).

**Table 1:** Demographic and clinical characteristics of the participants according to ginseng intake status.

	Overall	No ginseng intake	Ginseng intake	*p*
*n*	160	80	80	
Age, *y*	72.53 (5.88)	72.54 (6.27)	72.51 (5.51)	0.979^a^
Female, *n* (%)	111 (69.38)	54 (67.50)	57 (71.25)	0.607^b^
Education, year	9.79 (4.44)	9.33 (4.67)	10.25 (4.18)	0.189^a^
APOE4 status				0.845^b^
Negative	127 (79.37)	63 (78.75)	64 (80.00)	
positive	33 (20.63)	17 (21.25)	16 (20.00)	
Clinical diagnosis, MCI, *n* (%)	67 (41.88)	37 (61.67)	30 (37.50)	0.262^b^
VRS	23.54 (18.26)	24.58 (17.18)	22.50 (19.32)	0.472^a^
GDS score	10.81 (7.20)	10.55 (7.40)	11.06 (7.03)	0.654^a^
Annual income, *n* (%)				0.692^b^
<MCL	21 (13.13)	11 (13.75)	10 (12.50)	
≥MCL, <2 × MCL	49 (30.62)	22 (27.50)	27 (33.75)	
≥2 × MCL	90 (56.25)	47 (58.75)	43 (53.75)	
Alcohol drink status, *n* (%)				0.451^b^
Never	83 (51.87)	38 (47.50)	45 (56.25)	
Former	28 (17.50)	14 (17.50)	14 (17.50)	
Drinker	49 (30.63)	28 (35.00)	21 (26.25)	
Smoking status, *n* (%)				0.015^c^
Never	119 (74.38)	55 (68.75)	64 (80.00)	
Former	34 (21.25)	18 (22.50)	16 (20.00)	
Smoker	7 (4.37)	7 (8.75)	0 (0.00)	
Decrease in food intake over the past 3 months, *n* (%)	11 (6.88)	6 (7.50)	5 (6.25)	0.755^b^
Malnutrition, *n* (%)	0 (0.00)	0 (0.00)	0 (0.00)	
*Dietary pattern including food types*				
Protein, *n* (%)				0.178^b^
High	22 (13.75)	41 (51.25)	35 (43.75)	
Moderate	62 (38.75)	32 (40.00)	30 (37.50)	
Low	76 (47.50)	7 (8.75)	15 (18.75)	
Fruit & Vegetables, *n* (%)				0.405^b^
High	105 (66.62)	50 (62.50)	55 (68.75)	
Low	55 (34.38)	30 (37.50)	25 (31.25)	
*Serum nutritional markers*				
Albumin	4.58 (0.26)	4.57 (0.28)	4.58 (0.24)	0.831^a^
Glucose, fasting	107.89 (20.60)	108.01 (16.47)	107.77 (24.14)	0.942^b^
HDL-Cholesterol	54.58 (12.31)	56.05 (12.58)	53.11 (11.93)	0.134^a^
LDL-Cholesterol	96.55 (33.94)	96.40 (36.96)	96.72 (30.86)	0.950^a^
Ginseng type				<0.001^c^
Red	75 (46.88)	0 (0.00)	75 (93.75)	
White	5 (3.12)	0 (0.00)	5 (6.25)	
Ginseng intake, onset age, year				<0.001^b^
Midlife (< 65 years)	40 (25.00)	0 (0.00)	40 (50.00)	
Late life (≥ 65 years)	40 (25.00)	0 (0.00)	40 (50.00)	
Ginseng intake, duration, year				<0.001^b^
High use (≥ 5 years)	46 (28.75)	0 (0.00)	46 (57.50)	
Low use (< 5 years)	34 (21.25)	0 (0.00)	34 (42.50)	
*Cognition*				
Global cognitive score				
MMSE	25.66 (3.29)	25.36 (3.61)	25.95 (2.93)	0.260^a^
Memory score				
Immediate EMS	15.53 (3.92)	15.03 (4.08)	16.03 (3.70)	0.106^a^
Delayed EMS	19.83 (5.96)	18.66 (6.43)	21.00 (5.24)	0.013^a^
Non-memory score				
NMS	34.21 (6.34)	33.78 (7.03)	34.65 (5.59)	0.385^a^

APOE4, apolipoprotein E ε4 allele; MCI, mild cognitive impairment; VRS vascular risk score; GDS, geriatric depression scale; PASE physical activity scale for the elderly; EMS, episodic memory score; NMS, non-memory score.

Data are expressed as mean (standard deviation), unless otherwise indicated.

a By student *t* test.

b By chi-square test.

c By fisher exact test.

### Association between ginseng intake and cognition

**Figure 1:**
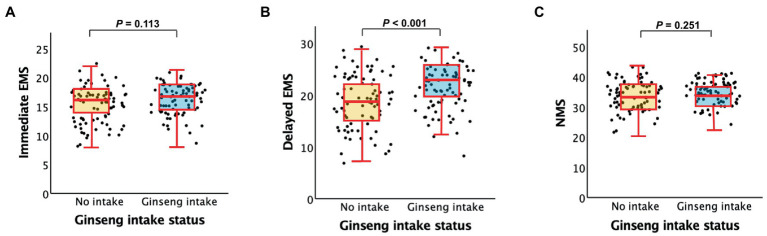
Box plots displaying ginseng intake status [(no ginseng intake vs. ginseng intake) and cognitive performances **(A)**. immediate EMS, **(B)**. delayed EMS, and **(C)**. NMS] in older subjects. EMS, episodic memory score; NMS, non-memory score. Multiple linear regression analyses were performed after adjusting for all potential covariates.

As shown in Table 2 and Figure 1, ginseng intake was associated with higher AD-specific cognition, i.e., delayed EMS compared to no ginseng intake, regardless of the model tested. By contrast, we did not find any significant difference in other cognition, i.e., immediate EMS and NMS between the ginseng intake groups.

**Table 2:** Results of the multiple linear regression analyses of the association between ginseng intake status and cognition.

	*B*	95% CI	*p*
**Memory score**			
Immediate EMS			
Model 1	0.695	−0.255 to 1.645	0.150
Model 2	0.787	−0.189 to 1.763	0.113
Delayed EMS			
Model 1	1.950	0.696 to 3.204	0.003
Model 2	2.182	0.926 to 3.438	<0.001
**Non-memory score**			
NMS			
Model 1	0.973	−0.753 to 2.698	0.267
Model 2	1.013	−0.726 to 2.752	0.251

APOE4, apolipoprotein E ε4 allele; EMS, episodic memory score; NMS, non-memory score; VRS vascular risk score; GDS, geriatric depression scale.

The first model included age, sex, APOE4, education, clinical diagnosis, VRS, GDS, and serum nutritional markers (albumin, glucose, HDL-cholesterol, LDL-cholesterol); the second model included age, sex, APOE4, education, clinical diagnosis, VRS, GDS, serum nutritional markers (albumin, glucose, HDL-cholesterol, or LDL-cholesterol), annual income, alcohol intake, smoking, and dietary pattern including food types (protein, fruits or vegetables).

### APOE4 status moderation of the association between ginseng intake and cognition

As shown in Table 3, the interaction between the ginseng intake and APOE4 status had a significant effect on the delayed EMS but neither the immediate EMS nor NMS. This indicates that APOE4 status has a moderating effect on the associations between the ginseng intake and delayed episodic memory cognition, but neither immediate episodic memory nor the NMS. As shown in Table 4, subgroup analyses according to APOE4 status showed that the ginseng intake was associated with a higher delayed EMS in the APOE4-negative but not the APOE4-positive subgroup.

**Table 3:** Results of multiple linear regression analyses that included interaction terms for the association between ginseng intake status and APOE4 status in predicting cognition.

	*B*	95% CI	*p*
**Memory score**			
Immediate EMS			
Ginseng intake	1.066	−0.008 to 2.141	0.052
APOE4 status	1.385	−2.438 to 5.208	0.475
Ginseng intake × APOE4 status	−1.484	−3.892 to 0.924	0.225
Delayed EMS			
Ginseng intake	3.144	1.538 to 4.749	<0.001
APOE4 status	2.327	−2.586 to 7.240	0.351
Ginseng intake × APOE4 status	−2.241	−3.463 to −1.018	<0.001
**Non-memory score**			
NMS			
Ginseng intake	1.380	−0.540 to 3.299	0.157
APOE4 status	2.655	−4.174 to 9.484	0.443
Ginseng intake × APOE4 status	−1.948	−6.249 to 2.352	0.372

APOE4, apolipoprotein ε4 allele; EMS, episodic memory score; NMS, non-memory score.

To explore the moderating effects of APOE4 status on the associations between ginseng intake status and cognition, i.e., immediate EMS, delayed EMS, and NMS, the multiple linear regression analyses were performed including two-way interaction terms between ginseng intake status and cognition as additional independent variables.

**Table 4:** Results of the multiple linear regression analyses of the association between ginseng intake status and cognition according to APOE4 subgroup.

	APOE4-negative			APOE4-positive		
	*B*	*95% CI*	*p*	*B*	95% CI	*p*
**Memory score**						
Immediate EMS						
Model 1	0.890	−0.191 to 1.971	0.106	−0.667	−3.204 to 1.870	0.589
Model 2	0.982	−0.111 to 2.076	0.078	−0.388	−3.297 to 2.521	0.779
Delayed EMS						
Model 1	2.250	0.947 to 3.552	<0.001	0.541	−3.291 to 4.374	0.771
Model 2	2.454	1.126 to 3.781	<0.001	1.430	−2.807 to 5.666	0.481
**Non-memory score**						
NMS						
Model 1	1.170	−0.748 to 3.087	0.229	2.091	−2.138 to 6.319	0.314
Model 2	1.261	−0.693 to 3.214	0.204	−0.037	−4.932 to 4.859	0.987

APOE4, apolipoprotein E ε4 allele; EMS, episodic memory score; NMS, non-memory score; VRS vascular risk score; GDS, geriatric depression scale.

The first model included age, sex, education, clinical diagnosis, VRS, GDS, and serum nutritional markers (albumin, glucose, HDL-cholesterol, LDL-cholesterol); the second model included age, sex, education, clinical diagnosis, VRS, GDS, serum nutritional markers (albumin, glucose, HDL-cholesterol, or LDL-cholesterol), annual income, alcohol intake, smoking, and dietary pattern including food types (protein, fruits or vegetables).

The interaction effect of ginseng intake and APOE4 status on delayed EMS was statistically significant (*B* = −2.241, *p* < 0.001), indicating that the association between ginseng intake and delayed EMS varied depending on APOE4 status. Specifically, the difference in delayed EMS associated with ginseng intake was significantly greater in the APOE4 negative group than in the APOE4 positive group. The negative B value reflects the direction of the interaction effect, where the relationship between ginseng intake and delayed EMS is positive (*B* = 3.244) in the APOE4 negative group but significantly less positive (*B* = 2.327) in the APOE4 positive group.

### Association between ginseng intake with (high vs. low) duration or (midlife vs. late life) onset and cognition

As shown in Tables 5, 6, ginseng intake was associated with higher delayed EMS in the high duration (≥ 5 years) and midlife onset (< 65 years) groups.

**Table 5:** Results of the multiple linear regression analyses of the association between ginseng intake status (high duration vs. low duration) and cognition.

High duration (≥ 5 years) (*n* = 46)	*B*	95% CI	*p*
**Memory score**			
Immediate EMS			
Model 1	0.803	−0.360 to 1.966	0.174
Model 2	0.854	−0.326 to 2.034	0.154
Delayed EMS			
Model 1	1.929	0.395 to 3.463	0.014
Model 2	2.141	0.595 to 3.688	0.007
**Non-memory score**			
NMS			
Model 1	1.140	−1.036 to 3.317	0.301
Model 2	1.060	−1.110 to 3.229	0.335
Low duration (< 5 year) (*n* = 34)	*B*	95% CI	*p*
**Memory score**			
Immediate EMS			
Model 1	0.142	−1.113 to 1.398	0.823
Model 2	0.032	−1.251 to 1.314	0.961
Delayed EMS			
Model 1	1.536	−0.107 to 3.180	0.067
Model 2	1.517	−0.150 to 3.183	0.074
**Non-memory score**			
NMS			
Model 1	0.684	−1.466 to 2.833	0.529
Model 2	0.543	−1.618 to 2.704	0.619

APOE4, apolipoprotein E ε4 allele; EMS, episodic memory score; NMS, non-memory score; VRS vascular risk score; GDS, geriatric depression scale.

For these analyses, we used the no ginseng intake (*n* = 80) as the reference.

The first model included age, sex, APOE4 status, education, clinical diagnosis, VRS, GDS, and serum nutritional markers (albumin, glucose, HDL-cholesterol, LDL-cholesterol); the second model included age, sex, APOE4 status, education, clinical diagnosis, VRS, GDS, serum nutritional markers (albumin, glucose, HDL-cholesterol, or LDL-cholesterol), annual income, alcohol intake, smoking, and dietary pattern including food types (protein, fruits or vegetables).

**Table 6:** Results of the multiple linear regression analyses of the association between ginseng intake status (midlife onset vs. late life onset) and cognition.

Midlife onset (< 65 years) (*n* = 40)				*B*	95% CI	*p*
**Memory score**						
Immediate EMS						
Model 1				0.742	−0.475 to 1.960	0.229
Model 2				0.837	−0.440 to 2.114	0.196
Delayed EMS						
Model 1				2.184	0.508 to 3.859	0.011
Model 2				2.617	0.921 to 4.314	0.003
**Non-memory score**						
NMS						
Model 1				1.328	−0.893 to 3.550	0.238
Model 2				1.279	−0.918 to 3.476	0.251
Late life onset (≥ 65 year) (*n* = 40)				*B*	95% CI	*p*
**Memory score**						
Immediate EMS						
Model 1				0.413	−0.808 to 1.635	0.504
Model 2				0.249	−0.985 to 1.483	0.690
Delayed EMS						
Model 1				1.448	−0.062 to 2.958	0.060
Model 2				1.290	−0.242 to 2.823	0.098
**Non-memory score**						
NMS						
Model 1				0.430	−1.672 to 2.532	0.686
Model 2				0.291	−1.854 to 2.437	0.788

APOE4, apolipoprotein E ε4 allele; EMS, episodic memory score; NMS, non-memory score; VRS vascular risk score; GDS, geriatric depression scale.

For these analyses, we used the no ginseng intake (*n* = 80) as the reference.

The first model included age, sex, APOE4 status, education, clinical diagnosis, VRS, GDS, and serum nutritional markers (albumin, glucose, HDL-cholesterol, LDL-cholesterol); the second model included age, sex, APOE4 status, education, clinical diagnosis, VRS, GDS, serum nutritional markers (albumin, glucose, HDL-cholesterol, or LDL-cholesterol), annual income, alcohol intake, smoking, and dietary pattern including food types (protein, fruits or vegetables).

### Sensitivity analyses

The results of the sensitivity analysis of subjects with MCI and those without a decrease in food intake over the past 3 months were similar for the delayed EMS, immediate EMS, and NMS (Table 7).

**Table 7:** Results of the multiple linear regression analyses of the association between ginseng intake status and cognition in participants with **(A)** MCI and **(B)** no decrease in food intake over the past 3 months.

(A)	Overall (*n* = 30)			APOE4-negative (*n* = 20)			APOE4-positive (*n* = 10)		
	*B*	95% CI	*p*	*B*	95% CI	*p*	*B*	95% CI	*p*
**Memory score**									
Immediate EMS									
Model 1	0.544	−1.227 to 2.316	0.540	1.319	−0.996 to 3.634	0.255	−0.732	−3.233 to 1.769	0.529
Model 2	0.456	−1.415 to 2.327	0.626	0.981	−1.475 to 3.437	0.421	−2.411	−8.312 to 3.491	0.342
Delayed EMS									
Model 1	2.923	0.337 to 5.508	0.027	4.018	1.216 to 6.821	0.006	−2.024	−7.764 to 3.716	0.450
Model 2	2.892	0.255 to 5.528	0.032	4.315	1.238 to 7.392	0.008	−3.116	−13.688 to 7.457	0.483
**Non-memory score**									
NMS									
Model 1	1.674	−1.432 to 4.780	0.285	2.515	−1.442 to 6.471	0.205	1.937	−3.854 to 7.729	0.473
Model 2	0.832	−2.416 to 4.079	0.609	2.339	−1.877 to 6.555	0.266	−6.730	−15.937 to 2,476	0.119
(B)	Overall (*n* = 75)			APOE4-negative (*n* = 60)			APOE4-positive (*n* = 15)		
	*B*	95% CI	*p*	*B*	95% CI	*p*	*B*	95% CI	*p*
**Memory score**									
Immediate EMS									
Model 1	0.719	−0.220 to 1.658	0.132	0.944	−0.125 to 2.014	0.083	−0.667	−3.204 to 1.870	0.589
Model 2	0.735	−0.254 to 1.723	0.144	0.960	−0.150 to 2.071	0.089	−0.388	−3.297 to 2.251	0.779
Delayed EMS									
Model 1	1.790	0.484 to 3.096	0.008	2.041	0.688 to 3.395	0.003	0.541	−3.291 to 4.374	0.771
Model 2	2.028	0.698 to 3.358	0.003	2.184	0.782 to 3.586	0.003	1.430	−2.807 to 5.666	0.481
**Non-memory score**									
NMS									
Model 1	0.686	−1.135 to 2.508	0.457	0.771	−1.273 to 2.815	0.456	2.091	−2.138 to 6.319	0.314
Model 2	0.648	−1.224 to 2.520	0.684	0.740	−1.383 to 2.862	0.491	−0.037	−4.932 to 4.859	0.987

MCI, mild cognitive impairment; APOE4, apolipoprotein E ε4 allele; EMS, episodic memory score; NMS, non-memory score; VRS vascular risk score; GDS, geriatric depression scale.

For these analyses, we used the no ginseng intake ([A] *n* = 37, 25, or 12 and [B] 74. 57, or 17 in overall, APOE4-negative, and APOE4-positive subgroups, respectively) as the reference.

The first model included age, sex, APOE4 status, education, clinical diagnosis, VRS, GDS, and serum nutritional markers (albumin, glucose, HDL-cholesterol, LDL-cholesterol); the second model included age, sex, APOE4 status, education, clinical diagnosis, VRS, GDS, serum nutritional markers (albumin, glucose, HDL-cholesterol, or LDL-cholesterol), annual income, alcohol intake, smoking, and dietary pattern including food types (protein, fruits or vegetables).

## Discussion

This study of non-demented older adults found that ginseng intake had a benefit on AD-specific cognition, i.e., the delayed episodic memory. It also showed that APOE4 status had a moderating effect on the association between ginseng intake status and delayed episodic memory cognition, and that ginseng intake had a benefit on the delayed episodic memory cognition in APOE4-negative but not APOE4-positive subjects. This may reflect interactions among ginseng intake, APOE4 status, and AD or related cognitive decline.

This study found that the benefit of ginseng intake was observed mainly on memory, not on non-memory, and it was more prominent in delayed episodic memory than immediate episodic memory. The earliest cognitive change in patients who will develop AD is episodic memory decline, particularly in delayed episodic memory decline (Howieson et al., 1997; Grober et al., 2000; Laakso et al., 2000; Bäckman et al., 2001, 2005; Tromp et al., 2015). Therefore, our results suggest that ginseng intake acts mostly on delayed episodic memory decline and may play a clinically significant role in early AD-related cognitive decline.

Previously, neuroprotective effects of ginseng on the pathological and clinical process of AD was reported, which may explain the mechanism underlying the relationship between ginseng intake and early AD-related cognitive decline (Razgonova et al., 2019; Li et al., 2021; Wang and Liu, 2023). In cell and animal models, ginseng acted on AD by regulating multiple signaling pathways, such as, the phosphatidylinositol 3-kinase/protein kinase B, adenosine-monophosphate activated-protein kinase/mammalian target of rapamycin, and nuclear factor kappa B pathways, in order to block or improve pathological processes, such as Aβ accumulation, tau phosphorylation, neuroinflammation, neurotrophic factors, apoptosis, and mitochondrial dysfunction in AD (Razgonova et al., 2019; Li et al., 2021; Wang and Liu, 2023). One preclinical study using an AD model revealed that ginseng treatment significantly improved learning and memory performance at 14 months of age and reduced brain senile plaques at this age (Qiu et al., 2014). *In vitro* experiments also found that ginseng treatment reduced Aβ 40 and 42 concentrations (Qiu et al., 2014). Another preclinical study in a transgenic mouse AD model found that long-term ginseng treatment attenuated Aβ deposition and short-and long-term memory impairment (Hwang et al., 2012). Clinical trials found that ginseng significantly improved working memory and attention in healthy young adults (Bell et al., 2022), global cognition and executive function in subjects with subjective memory impairment (Lee et al., 2022), visual memory function in MCI patients (Park et al., 2019), and global cognition in AD patients (Lee et al., 2008; Heo et al., 2012). Based on the preclinical findings, ginseng improves cognitive features of AD patients *via* AD pathologies, i.e., Aβ accumulation and tau phosphorylation. In human, however, the precise mechanism of ginseng in the pathologies of AD in the prevention and treatment of AD remain unclear.

The present study found that APOE4 status modulated the relationship between ginseng intake and delayed episodic memory cognition. A significant association between ginseng intake and delayed episodic memory cognition was apparent in subjects without APOE4, but not those with APOE4. We hypothesize that the benefit of ginseng may be masked in the latter subjects because APOE4 triggers the blood–brain barrier (BBB) dysfunction that predicts cognitive decline (Montagne et al., 2020). Also, Aβ clearance may differ by the APOE isoform, and could thus be affected by ginseng intake. APOE4 stimulates Aβ clearance less effectively than do APOE3 and APOE2; APOE4 status (compared to non-APOE4 status) may significantly inhibit Aβ removal (Wildsmith et al., 2013). Therefore, the protective effect of ginseng on AD-related cognition is apparent only in the APOE4-negative group because the benefit of ginseng is offset by the APOE4 affects the shared Aβ clearance pathway (Kurz and Perneczky, 2011).

The present study also found that ginseng intake was associated with a higher delayed EMS in the high duration (≥ 5 years) and midlife onset (< 65 years) groups. Regarding the high duration effect of ginseng on delayed EMS, two studies were similar to ours. A randomized open-label study indicated that long-term ginseng treatment for up to 2 years improved the cognitive deficit in AD patients (Heo et al., 2011). A population-based prospective cohort study of older Koreans also revealed that the high-use group (≥ 5 years) had a higher global cognitive score than the no-use group, which means that ginseng use for longer than 5 years may benefit cognitive function in late life (Lho et al., 2018). Regarding the effect of ginseng intake with midlife onset on delayed EMS, a human study supported our finding of a highly significant association between increasing age and decreased Aβ turnover rates, i.e., 2.5-fold longer half-life over five decades of age (Patterson et al., 2015). These changes in Aβ kinetics associated with aging present opportunities for treatment strategies, such as ginseng as an early intervention in middle age rather than old age.

## Limitations

Our study had several limitations. First, as it was cross-sectional in its design, causal relationships could not be inferred, which would require long-term prospective studies. Second, it did not measure brain AD pathology biomarkers, e.g., Aβ, tau, and APOE4-related metabolic biomarkers. These should be analyzed in future studies of the link between the ginseng intake and AD or related cognitive decline in APOE-negative older adults. Third, there was a possibility of recall biases in the ginseng intake history. Therefore, we evaluated the ginseng intake history in non-demented older adults using intensive interviews by researchers, not a questionnaire. Furthermore, information accuracy was ensured by interviewing reliable informants. Finally, the association between ginseng intake and AD or related cognitive decline may vary by ginseng dose. Two clinical trials assessed the dose-effects of ginseng on physical (Lee et al., 2018) and cognitive (Kennedy et al., 2001) performances; high-dose ginseng improved performance. However, in both studies, young (not older) adults received ginseng short-term. Ginseng efficacy may be affected by all of dose, administration period, and subject age. However, we could not measure the ginseng doses because commercial products do not consistently or clearly indicate the active ginseng levels. Long-term studies delivering defined ginseng doses to older adults are needed.

## Conclusion

Our study of older adults with no dementia suggests that ginseng intake (with high duration and midlife onset) had a benefit on AD-specific cognitive decline, i.e., the delayed episodic memory. In addition, APOE4 status moderates the association between ginseng intake status and AD-specific cognitive decline. Thus, APOE4 status could be considered a key component in the design of studies of AD and related cognitive decline that include ginseng intake as a preventive strategy.

## Data availability statement

The study data are not freely accessible because Institutional Review Board of the Hallym University Dongtan Sacred Heart Hospital prevents public sharing of such data for privacy reasons. However, the data are available on reasonable request after Institutional Review Board approval. Requests for data access can be submitted to an independent administrative coordinator by e-mail (yoon4645@gmail.com).

## Ethics statement

The studies involving human participants were reviewed and approved by Institutional Review Board of the Hallym University Dongtan Sacred Heart Hospital. The patients/participants provided their written informed consent to participate in this study.

## Author contributions

JK conceived, designed the study, served as principal investigator, and supervised the study. BL, YC, G-HS, I-GC, HK, JH, DY, and JK were involved in acquisition, or analysis and interpretation of the data and helped to draft the manuscript. BL, YC, G-HS, I-GC, HK, JH, DY, and JK were major contributors in writing the manuscript and critically revising the manuscript for intellectual content. All authors contributed to the article and approved the submitted version.

## Funding

This study was supported by the grants from the 2021 Korean Society of Ginseng. The funding sources had no role in the study design, data collection, data analysis, data interpretation, writing of the manuscript, or decision to submit it for publication.

## Conflict of interest

The authors declare that the research was conducted in the absence of any commercial or financial relationships that could be construed as a potential conflict of interest.

## Publisher’s note

All claims expressed in this article are solely those of the authors and do not necessarily represent those of their affiliated organizations, or those of the publisher, the editors and the reviewers. Any product that may be evaluated in this article, or claim that may be made by its manufacturer, is not guaranteed or endorsed by the publisher.
